# The influence of patient-centered communication on psychological distress: the chain mediating role of health-related self-efficacy and healthy lifestyle behaviors and the moderating role of social media use

**DOI:** 10.3389/fpsyt.2025.1562414

**Published:** 2025-07-14

**Authors:** Siying Angus Gong, Song Harris Ao, Yue Selene Chen

**Affiliations:** ^1^ Guangdong University of Finance and Economics, Guangzhou, China; ^2^ School of Journalism and Communication, Sun Yat-sen University, Guangzhou, China; ^3^ Faculty of Social Sciences, University of Macau, Macao, Macao SAR, China

**Keywords:** patient-centered communication, psychological distress, health-related self efficacy, healthy lifestyle behaviors, social media use

## Abstract

**Background:**

Psychological distress remains a pervasive issue, influenced by complex psychosocial and behavioral mechanisms. Nevertheless, there is a paucity of research exploring how patient-centered communication influences psychological health through intermediary mechanisms, especially the sequential roles played by health-related self-efficacy and healthy lifestyles.

**Method:**

This study was conducted on de-identified public data from the Health Information National Trends Survey 2022 (HINTS 6), with a sample size of 6,252. It first proposed and tested a chain mediation model linking patient-centered communication and psychological distress, mediated by health-related self-efficacy and healthy lifestyle behaviors. Based on the model, it explored the moderating role of social media use in this model.

**Result:**

This study found that patient-centered communication is negatively associated with psychological distress and health-related self-efficacy and healthy lifestyle behavior play a chain mediating role between them. Moreover, social media use enhances the positive impact of patient-centered communication on health-related self-efficacy.

**Conclusion:**

This study demonstrates the generalizability of patient-centered communication in alleviating psychological distress through the chain mediating pathway of health-related self-efficacy and healthy lifestyle behaviors. Notably, it reveals the moderating effect thresholds of social media use on this pathway. These findings provide both theoretical foundations and practical guidelines for: (1) optimizing doctor-patient communication strategies; (2) media literacy interventions under the WHO health literacy framework; and (3) improving psychological support in post-pandemic telemedicine.

## Introduction

Psychological distress has become an increasingly serious public health issue. According to statistics from the WHO, approximately 264 million people worldwide suffer from psychological disorders (1). Psychological distress is a state of emotional suffering caused by stressors and demands that are difficult to cope with in daily life (2, 3), manifested as emotional instability and even psychoneurosis. Psychological distress affects an individual’s quality of life and work efficiency and increases the risk of developing other chronic diseases. Existing studies have shown that psychological distress is closely related to the occurrence and progression of various chronic diseases, including cardiovascular and immune system disorders, even at low to moderate levels of distress. For example, a persistent state of psychological distress may lead to elevated blood pressure and an increased heart rate, thereby raising the likelihood of cardiovascular disease (4); moreover, the suppressive effect of psychological distress on the immune system may make the body more susceptible to various pathogens, thereby increasing the risk of illness (5). Therefore, paying attention to psychological distress and seeking effective interventions is of great practical significance.

In contemporary healthcare, patient-centered communication (PCC) is widely recognized as a critical factor in improving health outcomes by fostering trust, enhancing understanding, and promoting patient engagement. Multiple studies have established that when clinicians engage in patient-centered communication, patients tend to report higher satisfaction and a better perception of the quality of care provided (6, 7). Patient-centered communication is pivotal for overcoming barriers related to health literacy and cultural discordance (7–9). However, despite its recognized benefits, the psychological distress experienced by patients remains a pervasive issue, influenced by complex psychosocial and behavioral mechanisms. While existing research has explored the direct effects of PCC on psychological health (10), the underlying pathways—particularly the sequential roles of health-related self-efficacy and healthy lifestyle—remain underexamined. Furthermore, in an era of digital connectivity, social media use may serve as a key moderator in this relationship, potentially amplifying or mitigating the effects of PCC on psychological well-being.

This study seeks to bridge these gaps by investigating the chain mediating roles of health-related self-efficacy and healthy lifestyle in the relationship between PCC and psychological distress, while also examining how social media use moderates these dynamics. By integrating theories from health communication, social cognitive theory, and behavioral medicine, this research aims to provide a nuanced understanding of how effective patient-provider interactions translate into improved mental health outcomes. The findings will offer valuable insights for healthcare practitioners, policymakers, and digital health designers seeking to optimize communication strategies and leverage technology to reduce patients’ psychological distress.

### Hypothesis

Health-related self-efficacy and healthy lifestyle behaviors, as important psychological and behavioral factors, may serve as mediators between patient-centered communication and psychological distress. Health-related self-efficacy, rooted in Albert Bandura’s concept of self-efficacy, refers to an individual’s confidence and ability to successfully adopt healthy behaviors and maintain a healthy state (11). Health-related self-efficacy extends the concept of self-efficacy specifically to the domain of health management and behavioral change. An existing study demonstrates that health-related self-efficacy partially mediated the relationship between patient-centered communication and psychological distress among ovarian cancer patients, and in a broader cancer population, self-efficacy along with cognitive reappraisal fully mediated this association (12). This finding underscores that effective communication can empower patients to manage their conditions more effectively, thereby reducing negative emotional outcomes through enhanced self-efficacy.

Beyond its impact on psychological well-being, health-related self-efficacy is intricately linked to healthy lifestyle behaviors. Research has shown that higher health-related self-efficacy serves as a key predictor of successful post-treatment outcomes (13), smoking cessation (14), dieting, and weight control (13), highlighting its importance in shaping sustainable health behaviors and long-term well-being. An individual’s confidence in their health, disease treatment, and postoperative recovery can also effectively bring about positive changes in their mood and psychological state (2, 15, 16). Health-related self-efficacy becomes increasingly significant as patients transition from contemplating behavioral change to actively modifying their lifestyles (17). For example, numerous studies have confirmed that physical activity has a positive impact on psychological distress, helping to alleviate negative emotions such as distress, anxiety, and depression (4, 5). Moderate-intensity physical activity can stimulate the secretion of “happy hormones,” such as serotonin, endorphins, and dopamine, thereby can act by reducing levels of anxiety, depression, and other forms of psychological distress (18, 19). Complementary to these findings, it is elucidated that interventions based on patient motivation and effective communication strategies, such as motivational interviewing, enhance health-related self-efficacy and promote healthier lifestyle behaviors (20). Such approaches facilitate patients’ engagement in self-care activities, contributing to improved mental health outcomes.

Further support for the mediating role of health-related self-efficacy is provided by intervention studies in chronic disease settings. It is indicated that structured patient empowerment programs designed to enhance self-efficacy and modify illness perceptions can lead to reductions in psychological distress (21). Theoretical models based on social cognitive theory suggest that confidence in one’s ability to enact behavior (self-efficacy) is crucial for translating communicative efforts into productive health behaviors, which subsequently mitigate psychological distress (22). Collectively, these findings suggest that patient-centered communication, by bolstering patients’ confidence in managing their health, indirectly promotes the adoption of healthy lifestyle behaviors and reduces distress.

Additionally, patients who experience patient-centered communication are more likely to internalize the rationale for change, resulting in improvements in self-efficacy and adherence to healthier lifestyle practices. Patient-centered communication is recognized as a critical determinant in promoting healthy lifestyle behaviors by enhancing health-related self-efficacy (23, 24). Research among patients with chronic conditions has demonstrated that patient-centered care is positively associated with healthy lifestyle behaviors, which in turn improve health-related quality of life (25). Similarly, studies in clinical settings have shown that PCC intervention strategies—such as those inspired by the 5A model—facilitate efficient counseling for lifestyle behavioral change, contributing to cardiovascular health improvement (26). Additionally, findings among cancer survivors indicate that effective communication increased patient trust and self-efficacy, leading to more active engagement in healthy behaviors (27).

Health-related self-efficacy appears to be a pivotal mechanism through which patient-centered exerts its influence. In clinical contexts, enhanced provider-patient communication has been shown to foster patients’ confidence in managing their conditions, as evidenced by improved self-care behaviors in chronic diseases such as type 2 diabetes (24); and reduced decisional conflicts in treatment decisions (28). In the digital health context, online health community engagements and telehealth interventions have leveraged PCC principles to provide both informational and emotional support, thereby enhancing patients’ health-related self-efficacy and promoting sustainable lifestyle changes (29). Moreover, research in older adult and chronic disease populations indicates that even modest enhancements in self-efficacy can lead to notable reductions in psychological distress (12, 21). Similarly, interventions targeting improved communication have been linked with increased patient self-management and adherence to health-promoting behaviors, which may act as protective factors against the negative effects of psychological distress (17, 20). Collectively, these studies suggest that enhancing self-efficacy and engaging in healthy lifestyle behaviors are viable mechanisms through which patient-centered communication can alleviate psychological distress. Intervention studies suggest that when social media is used as a medium for health campaigns and interactive interventions, improvements in self-efficacy are often observed (30, 31).

The interplay between patient-centered communication and health-related self-efficacy is complex, and emerging evidence suggests that social media use may moderate this relationship. Specifically, social media platforms can enhance the exchange and reinforcement of information shared during clinical interactions, potentially amplifying the effect of patient-centered communication on patients’ self-efficacy and overall empowerment in health management (32). Social media provides patients with supplemental information and fosters a community environment that encourages sharing experiences, which can further enhance confidence in managing health issues (33, 34). In this way, social media extends the principles of patient-centered communication—such as dialogue, personalization, and empathic engagement—beyond traditional clinical settings (35). Empirical studies demonstrate that when healthcare providers engage with patients on social media, it can bridge social, economic, and geographic gaps, complementing in-person patient-centered communication interactions and reinforcing health-related behavioral intentions (32, 34). Existing research found that prior positive experiences with health information obtained through social media enhance self-efficacy, indicating that the relationship between patient-centered communication and health-related self-efficacy is more potent among patients with favorable histories of online health engagement. Furthermore, the synergistic impact of social media use and patient-centered communication on health-related self-efficacy appears to rely on patient-specific factors such as prior experience and overall health literacy. Patients who regard social media as a trusted source of health-related information are more likely to internalize messages conveyed during patient-centered interactions, which can lead to improved self-management behaviors and greater overall efficacy (34). The evidence supports a conceptual model where social media acts as a moderator by providing additional layers of social support, thereby strengthening the positive outcomes of patient-centered communication on health-related efficacy.

**Figure 1:**
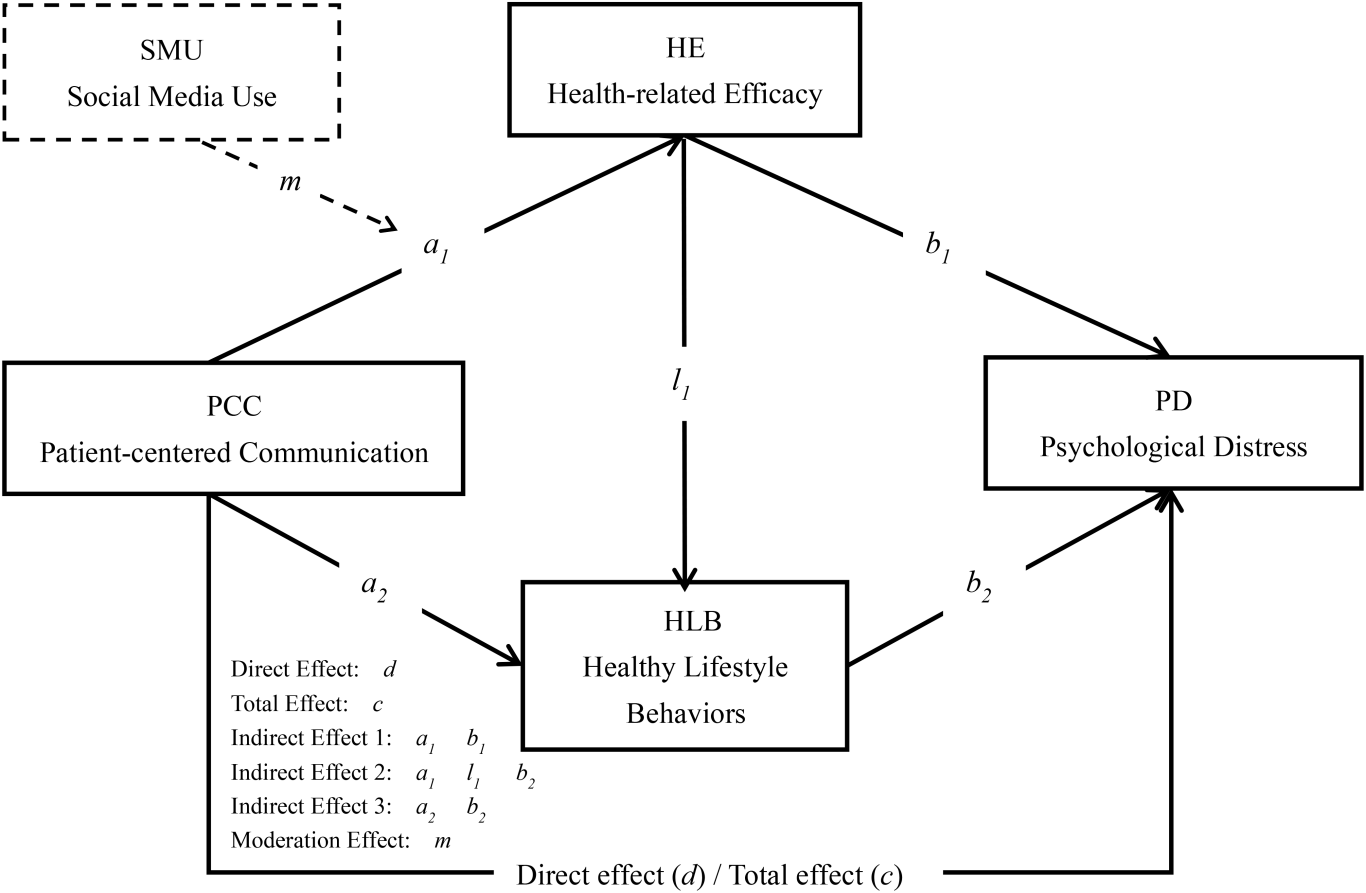
Pathways between PCC and PD.

Existing research has explored the impact of patient-centered communication on individual psychological distress, the role of health-related self-efficacy and healthy lifestyle behaviors in psychological distress, as well as the association between social media use and health-related self-efficacy. However, few studies have combined these factors to investigate the chain mediation effects of health-related self-efficacy and healthy lifestyle behaviors in the mechanism through which patient-centered communication affects psychological distress, and the moderation effects of social media use in the relationship between patient-centered communication and health-related self-efficacy. Therefore, this study will further explore the impact mechanism of patient-centered communication on psychological distress through empirical research, analyze the chain mediation effects of health-related self-efficacy and healthy lifestyle behaviors and the moderating effect of social media use. Thus, the following direct and indirect relationships (see **Figure 1**) were proposed:

H1: patient-centered communication is positively related to psychological distress.
H2: Health-related self-efficacy mediates the relationship between patient-centered communication and psychological distress.
H3: Healthy lifestyle behaviors mediate the relationship between patient-centered communication and psychological distress.
H4: Health-related self-efficacy and healthy lifestyle behaviors sequentially mediate the relationship between patient-centered communication and psychological distress.
H5: Social media use moderates the relationship between patient-centered communication and health-related self-efficacy.

The study will provide a theoretical basis for the development of effective psychological health interventions, while also offering practical guidance for the promotion of patient-centered communication and improving public psychological health, encouraging effective patient-centered communication and social media use to improve psychological health issues.

### Methods

This study was tested using SPSS 24.0 and PROCESS (36). It employed hierarchical multiple regression analysis (controlling for demographic variables before sequentially introducing independent and mediating variables) coupled with Bootstrap sampling (N=5,000) to examine the chain-mediating effects of health-related self-efficacy and healthy lifestyle behaviors. Subsequently, interaction-term regression analysis (patient-centered communication × social media use) was conducted to test the moderating effects. In this study, we introduce Min-Max normalization (37) as a complementary technique to compare the estimates of all paths in the mediation model. All research variables were converted into a common measurement scale of 0 to 1. It referred to the regression coefficients generated by Min-Max normalization as percentage coefficients (bp) (38). Finally, the key results of this study are presented in the form of percentage coefficients (bp), standardized coefficients (β), along with a 95% confidence interval (95%Boot CI) and p value.

### Study design and sampling

The data for this study came from the Health Information National Trends Survey administered by the National Cancer Institute (HINTS 6, http://hints.cancer.gov/). The data used in this study were collected from March through November 2022, with a sample size of 6,252. Univariate information of key variables, including psychological distress, patient-centered communication, health-related self-efficacy, healthy lifestyle behaviors, and social media use was shown in **Table 1**.

**Table 1:** Descriptive statistics of focal variables (N = 6,252).

Psychological distress (PD)^1^ Cronbach’s α =0.862		Nearly every day	More than half the days	Several days	Not at all		NV
Hints 6 H11.Over the past 2 weeks, how often have you been bothered by any of the following problems? (%)							
1. little interest or pleasure in doing things.		5.8	9.2	21.1	59.2		4.6
2. feeling down, depressed or hopeless.		3.1	6.4	21.9	63.9		4.7
3. feeling nervous, anxious, or on edge.		4.9	7.8	27.1	55.3		4.8
4. not being able to stop or control worrying.		5.2	7.5	22.4	60.3		4.6

Patient-centered communication (PCC)^2^ Cronbach’s α = 0. 934		Never	Sometimes	Usually	Always		NV
Hints 6 C3. Respondents were asked to answer: How often did they do each of the following? (%)							
1. give you the chance to ask all the health-related questions you had		1.1	8.1	27.1	50.8		12.9
2. give the attention you needed to your feelings and emotions.		3.3	15.2	29.9	38.3		13.3
3. involve you in decisions about your health care as much as you wanted.		1.9	10.6	28.5	45.8		13.1
4. make sure you understood the things you needed to do to take care of your health.		1.3	8.6	27.2	49.8		13.1
5. explain things in a way you could understand.		1.0	7.0	27.5	51.5		13.0
6. spend enough time with you.		3.4	16.6	30.0	36.5		13.4
7. help you deal with feelings of uncertainty about your health or health care.		4.8	16.8	29.7	34.9		13.7

Health-related self-efficacy (HE)^3^	Not confident at all	A little confident	Somewhat confident	Very confident	Completely confident		NV
Hints 6 H2. Overall, how confident are you about your ability to take good care of your health? (%)	1.2	4.1	22.4	42.5	26.3		3.5

Health lifestyle behaviors (HLB)^4^					0	1	NV
Health lifestyle behaviors (HLB) is the mean of the responses of four questions, K1, L1, N4, L2, each coded 0 or 1.							
Hints 6 K1. In a typical week, do you do any physical activity or exercise of at least moderate intensity, such as brisk walking, bicycling at a regular pace, and swimming at a regular pace (do not include weightlifting)? (%)					23.2	71.6	5.2
Hints 6 L1. Think about the last time you ordered food in a fast food or sit down restaurant, did you notice calorie information listed next to the food on the menu or menu board? (%)					48.8	46.4	4.7
Hints 6 N4. Have you smoked at least 100 cigarettes in your entire life?					61.0	33.1	5.9
Hints 6 L2. During the past 30 days, did you have at least one drink of any alcoholic beverage? (%)					47.2	43.1	9.7

Social media use (SMU)^5^ Cronbach’s α = 0.684		Never	Less than once a month	A few times a month	At least once a week	Almost every day	NV
Hints 6 B12. In the past 12 months, how often did you do the following? (%)							
1. visited a social media site.		20.3	6.2	7.7	12.0	52.3	1.5
2. shared personal health information on social media.		82.5	9.7	2.7	1.2	0.9	2.9
3. shared general health-related information on social media (for example, a news article).		68.9	19.8	6.4	2.1	0.9	1.8
4. Interacted with people who have similar health or medical issues on social media or online forums.		77.5	12.5	4.8	2.2	1.2	1.8
5. watched a health-related video on a social media site (for example, YouTube).		42.9	29.4	16.7	6.7	2.7	1.5

^1^ Psychological distress: A state of emotional suffering characterized by symptoms of anxiety and depression, often stemming from stress or mental health challenges.

^2^ Patient-centered communication: A healthcare approach that prioritizes the patient’s needs, preferences, and values through empathetic, collaborative, and clear dialogue.

^3^ Health-related self-efficacy: An individual’s confidence in their ability to manage their health behaviors and overcome challenges to achieve wellness goals.

^4^ Health lifestyle behaviors: Daily actions and habits (e.g., diet, exercise ) that influence physical and mental well-being, either positively or negatively.

^5^ Social media use: The engagement with digital platforms (e.g., Facebook, Youtube) for communication, information-sharing, or entertainment, which can impact mental and social health.

### Data privacy and ethical consideration

This study utilized de-identified public data sourced from the Health Information National Trends Survey (HINTS). All primary privacy protections were implemented by the National Cancer Institute (NCI) via standardized procedures, including (1) the removal of direct identifiers, (2) the aggregation of rare demographic combinations, and (3) statistical disclosure control for geographic variables. The dataset conforms to stringent ethical standards and has been approved by the relevant institutional review board (IRB). Informed consent was obtained from all participants prior to data collection, and the study procedures adhere to established ethical guidelines and regulations. Notably, psychological distress measures have been rigorously anonymized in accordance with HIPAA Safe Harbor standards. As Zhang et al. (39) demonstrated, such processed public data achieve “functional anonymization” when researchers avoid high-risk combinations (e.g., distress scores + rare demographics). Our analysis protocol explicitly excludes these combinations, aligning with their Ethical Data Sharing Matrix recommendations for behavioral research.

## Measurement

### Dependent variable: psychological distress

Psychological distress measures the severity of psychological health problems. Participants were asked to indicate over the past 2 weeks, how often have they been bothered by any of the following problems: little interest or pleasure in doing things; feeling down, depressed, or hopeless; feeling nervous, anxious, or on edge; not being able to stop or control worrying. Each of the 4-item rates psychological distress using a 4-item Likert scale of 1 (not at all), 2 (several days), 3 (more than half the days), and 4 (nearly every day). By an average of the 4 items to evaluate the severity of psychological health problems.

### Independent variable: patient-centered communication

Patient-centered Communication (PCC) was accessed by 7 items. The same measurement was applied in studies related to cancer survivors’ experience, and e-health information exchange patterns (40–42). Respondents were asked to report during the past 12 months, how often their doctors, nurses, or other health professionals (1) gave them the chance to ask all the health-related questions they had; (2) gave the attention they needed to their feelings and emotions; (3) involve them in decisions about their health care as much as they wanted; (4) make sure they understood the things they needed to do to take care of their health; (5) explain things in a way they could understand; (6) spend enough time with them; (7) help them deal with feelings of uncertainty about their health or health care. The answer to each question was categorized by 4 scores (1 = “Never” to 4= “Always”). The higher score represented a higher level of patient-centered Communication.

### Mediators: health-related self-efficacy and healthy lifestyle behaviors

Health-related self-efficacy (HE) was used to test the individual perceptions of physician communicative behaviors. It was measured using one item, similar to previous studies (43). Respondents were asked to report overall, how confident are you about your ability to take good care of your health? A five-point Likert scale was used (1= “not confident at all” to 5= “completely confident”). The higher score indicated a higher level of cancer-related self-efficacy.

Health lifestyle behaviors measure respondents’ behaviors and habits related to health. The following four items are used by the HINTS to calculate the variable HL in this study, which were adapted from previously validated scales (44). Participants were asked to report (1) in a typical week, did they do any physical activity or exercise of at least moderate intensity; (2) during the past 30 days, did you have at least one drink of any alcoholic beverage; (3) when they ordered food in a fast food or sit down restaurant, did they notice calorie information listed next to the food on the menu or menu board; (4) have they smoked at least 100 cigarettes in their entire life? Dichotomous answers (1 = yes, 0 = no) were added up to construct the variable.

### Moderator: social media use

Social media use was measured by respondents’ behaviors using social media in the past 12 months. A five-point Likert scale was used 1= Never, 2= Less than once a month 3= A few times a month 4= At least once a week 5= Almost every day. The scales have been widely applied in prior research. Participants were asked to answer the following questions: In the past 12 months, how often did you do the following? (1) visited a social media site. (2) shared personal health information on social media. (3) shared general health-related information on social media (for example, a news article). (4) Interacted with people who have similar health or medical issues on social media or online forums. (5) watched a health-related video on a social media site (for example, YouTube).

### Control variables

Demographic variables were used as controls to reduce confounding effects. As shown in **Table 2**, Demographic variables included age, gender, education, and family annual income. Respondents were asked to answer what is their age? On their original birth certificate, were they listed as male or female? What is the highest grade or level of schooling they completed? (1= “Less than High School” 2= “High School Graduate” 3= “Some College” 4= “College Graduate or More”). Family Annual Income (1=“$0 to $9,999” 2=“$10,000 to $14,999”, 3=“$15,000 to $19,999”, 4=“$20,000 to $34,999”, 5=“$35,000 to $49,999” 6= “$50,000 to $74,999” 7=“$75,000 to $99,999” 8=“$100,000 to $199,999” 9=“$200,000 or more”.

**Table 2:** Demographic characteristics (N=6252).

Demographic characteristic	Frequencies (%)
Age	
Gender	
Male	2307 (36.9%)
Female	3535 (56.5%)
NV	98 (1.6%)
Education	
Less than 8 years	116 (1.9%)
8 through 11 years	271 (4.3%)
12 years or completed high school	1068 (17.1%)
Post high school training other than college	433 (6.9%)
Some college	1239 (19.8%)
College graduate	1613 (25.8%)
Postgraduate	1108 (17.7%)
NV	404 (6.5%)
Family annual income	
$0 to $9,999	389 (6.2%)
$10,000 to $14,999	304 (4.9%)
$15,000 to $19,999	266 (4.3%)
$20,000 to $34,999	729 (11.7%)
$35,000 to $49,999	732 (11.7%)
$50,000 to $74,999	937 (15.0)
$75,000 to $99,999	694 (11.1%)
$100,000 to $199,999	1012 (16.2%)
$200,000 or more	457 (7.3%)
NV	732 (11.7%)

### P value and effect size measures

*P <.05 for statistical acknowledgment*. *P <.05*, in this study, is regarded as a prescreen, passing which allows for analysis of the types and the sizes of the estimated effects. Therefore, this study refers to *P* = 0.05 as “statistically acknowledged” instead of “statistically significant” (38, 45, 46).

*Percentage coefficient (b_p_)*, a regression coefficient, is calculated when both independent and dependent variables are on 0–1 percentage scale (*p_s_*). Equation 1 (Eq. 1) can be used to transform variables that are not on a percentage scale into ones that are. Here, *s_p_* is the percentage score after transformation, *s_os_* is the original score, *s_cx_* is the conceptual maximum on the original scale, and *s_cn_* is the conceptual minimum on the original scale (38).


Eq. 1 $$s_{p}=\frac{s_{os}-s_{cn}}{s_{cx}-s_{cn}}$$

## Results

**Table 3** presents the correlation matrix displaying the associations among all variables in the study.

**Table 3:** Correlations.

Variables	PD	PCC	HE	HLB	SMU	Age	Gender	Education	Income
PD	1								
PCC	-0.184 ***	1							
HE	-0.309 ***	0.288 ***	1						
HLB	-0.137 ***	0.039 **	0.160 **	1					
SMU	0.025	0.031 **	0.055 ***	-0.014	1				
Age	-0.187 ***	0.093 ***	-0.007	-0.083 ***	-0.130 ***	1			
Gender	-0.076 ***	-0.005	-0.048 ***	-0.051 ***	-0.032 *	0.050 ***	1		
Education	-0.092 ***	0.016	0.117 ***	0.139 ***	-0.076 ***	-0.110 ***	0.032 *	1	
Income	-0.195 ***	0.060 ***	0.152 ***	0.095 ***	-0.010	-0.125 ***	0.134 **	0.481 ***	1

PD, Psychological distress; PCC, Patient-centered communication; HE, Health-related self-efficacy; HLB, Health lifestyle behaviors; SMU, Social media use.

* p < .05; ** p < .01; *** p < .001.

### Hypothesis testing: mediation effect

See **Table 1** and **Table 4** shows a significant direct association between patient-centered communication and psychological distress PD (b_p_ = -.1553, β = -.1473, 95%CI [-.1835, -.1271]. p <.001). Thus, H1 was supported.

**Table 4:** Mediation models.

Path	b_p_	β	SE	95%Boot CI	p
DV: Psychological distress (Model 1)					
PCC➔HE (a_1_ path)	0.2758	0.2803	0.0135	[0.2493, 0.3023]	0.0000
PCC➔HLB (a_2_ path)	0.0030	0.0028	0.0160	[-0.0284, 0.0345]	0.8505
HE➔HLB (l_1_ path)	0.1646	0.1495	0.0164	[0.1323, 0.1968]	0.0000
HE➔PD (b_1_ path)	-0.2681	-0.2502	0.0149	[-0.2973, -0.2390]	0.0000
HLB➔PD (b_2_ path)	-0.1075	-0.1104	0.0129	[-0.1329, -0.0821]	0.0000
PCC➔PD (direct effect, d path)	-0.0761	-0.0722	0.0143	[-0.1043, -0.0480]	0.0000
PCC➔PD (total effect, c path)	-0.1553	-0.1473	0.0144	[-0.1835, -0.1271]	0.0000
PCC➔HE➔ PD (indirect effect, a_1_ × b_1_)	-0.0740	-0.0701	0.0062	[-0.0863, -0.0622]	/
PCC➔HE➔ HLB➔ PD (indirect effect, a_1_ × b_2_ × l_1_)	-0.0049	-0.0046	0.0008	[-0.0066, -0.0034]	/
PCC➔HLB ➔ PD (indirect effect, a_2_ × b_2_)	-0.0003	-0.0003	0.0018	[-0.0039, 0.0033]	/

PD, Psychological distress; PCC, Patient-centered communication; HE, Health-related self-efficacy; HLB, Health lifestyle behaviors.

Note: p-values are not computed for bootstrapped indirect effects.

H2 predicted that health-related self-efficacy mediates the relationship between patient-centered communication and psychological distress. As depicted in **Table 4**, patient-centered communication was significantly and positively associated with health-related self-efficacy (b_p_ = .2758, β = .2803, 95%CI [.2493,.3023], p <.001). Meanwhile, health-related self-efficacy was negatively associated with psychological distress (b_p_ = -.2681, β = -.2502, 95%CI [-.2973, -.2390], p <.001). The indirect effect a_1_ × b_1_ (b_p_ = -.0740, β = -.0701, 95%CI [-.0863, -.0622] was statistically acknowledged. H2 was thus supported.

H3 predicted that healthy lifestyle behaviors mediate the relationship between patient-centered communication and psychological distress. **Table 4** showed that although a significant relationship between healthy lifestyle behaviors and psychological distress was acknowledged (b_p_ = -.1075, β = -.1104, 95%CI [-.1329, -.0821], p<.001), there was no significant direct association between patient-centered communication and healthy lifestyle behaviors (b_p_ = .0030, β = .0028, 95%CI [-.0284,.00345], p = .8505), with the nonsignificant indirect effect a_2_ × b_2_ (b_p_ = -.0003 β = -.0003, 95%CI [-.0039,.0033). Therefore, H3 was not supported.

H4 predicted that patient-centered communication is related to psychological distress through the serial mediation of health-related self-efficacy and healthy lifestyle behaviors. As shown in **Table 4**, the indirect effect a_1_ × b_1_ × l_1_ (b_p_ = -.0049, β = -.0046, 95%CI [-.0066, -.0034] between patient-centered communication and psychological distress, via sequential mediators of health-related self-efficacy and healthy lifestyle behaviors were statistically acknowledged, supporting H4. See **Table 4** for details of the main results.

### Hypothesis testing: moderation effect

**Figure 2:**
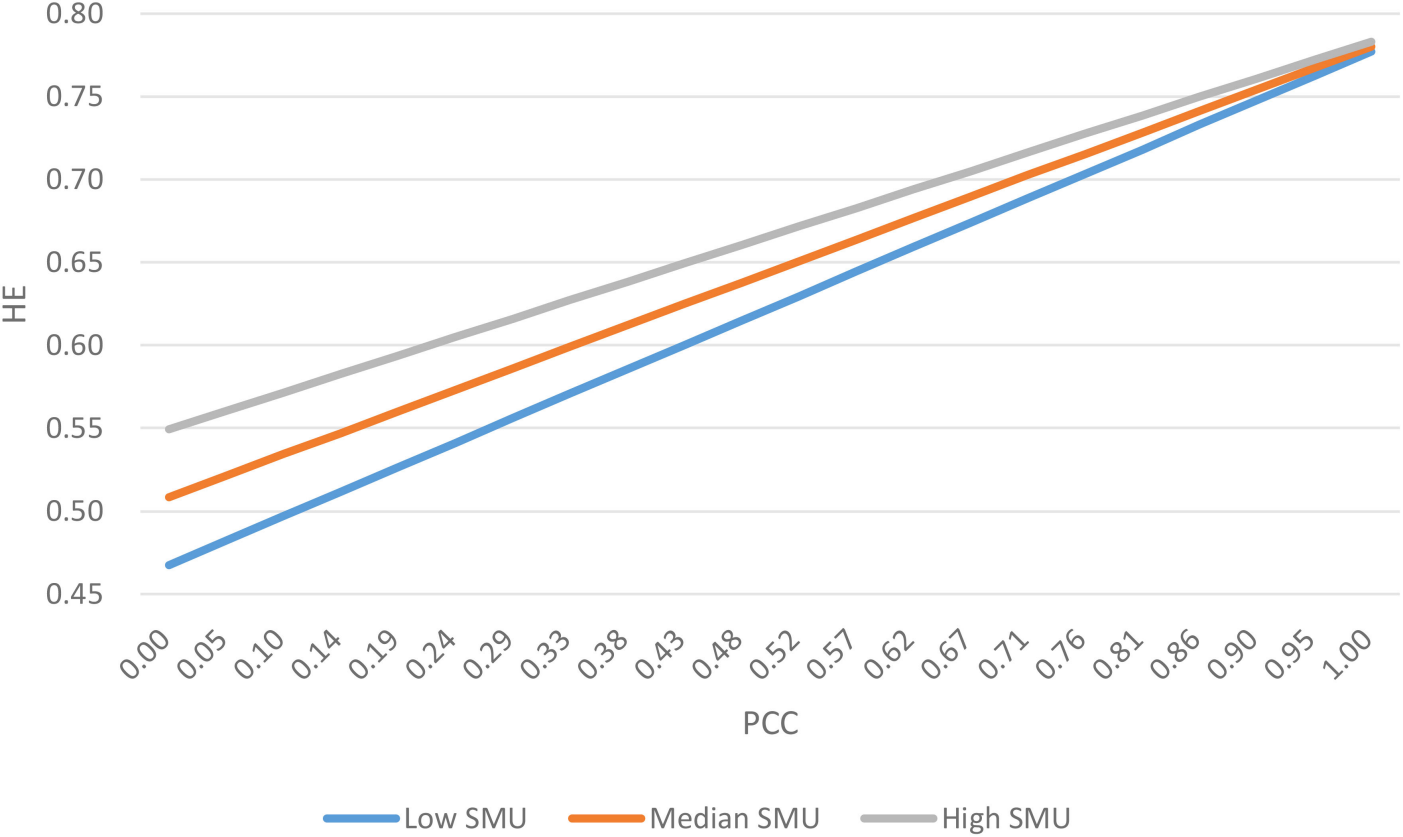
Moderating effect of SMU on PCC➔ HE direct path (*a_1_* path).

H5 predicted that social media use moderates the association between patient-centered communication and health-related self-efficacy. As shown in **Figure 2**, the slope for low social media use was the highest, followed by median and high social media use, suggesting that the effect of patient-centered communication on health-related self-efficacy was stronger for users with low social media use compared to those with middle or high social media use, thus supporting H5.

## Discussion

### Principle findings

This study used multiple regression analysis to explore the impact of patient-centered communication on psychological distress and its underlying mechanism, and reached the following conclusions:

Patient-centered communication is negatively associated with psychological distress, patient-centered communication is positively related to health-related self-efficacy, and health-related self-efficacy is positively related to healthy lifestyle behaviors. Both health-related self-efficacy and healthy lifestyle behaviors are negatively related to psychological distress.

The results of the current study show that health-related self-efficacy and healthy lifestyle behavior play a chain mediating role in the relationship between patient-centered communication and psychological distress. This means patient-centered communication can be used to promote individual psychological well-being through external and internal health-related self-efficacy building and healthy lifestyle behavior development.

However, the results of this study do not support the mediating role of healthy lifestyle behaviors in the relationship between patient-centered communication and psychological distress. This suggests that patient-centered communication cannot directly improve individuals’ health behavior. It can only enhance healthy lifestyle behaviors through health-related self-efficacy, and then improve psychological distress.

### Theoretical implication

This study deepened the overall understanding of the intrinsic link between patient-centered communication and psychological distress. Existing studies tend to explore the direct effects of patient-centered communication on psychological distress, but there is insufficient discussion on the internal mechanism of how patient-centered communication affects psychological distress. This paper provides empirical evidence of the chain mediating effect of health-related self-efficacy and healthy lifestyle behaviors and reveals the internal mechanism of the effect of patient-centered communication on psychological distress, which enriches the theoretical research on psychological health. The theoretical implication is similar to prior research indicating that when healthcare providers actively listen, tailor health messages, and incorporate patient values, patients are more likely to adhere to clinical recommendations and engage in self-management of chronic conditions (47–49). The integration of complementary tools such as motivational interviewing and mindfulness into clinical conversations further enhances health behavior improvement by enabling patients to resolve ambivalence and build the confidence necessary to make and sustain lifestyle behavior changes (47, 48). These behaviors include maintaining a healthy weight, engaging in regular physical activity, consuming a balanced diet rich in fruits and vegetables, abstaining from smoking, and moderating alcohol intake (50, 51).

Patient-centered communication employs techniques to enhance intrinsic motivation, and self-efficacy, and improve mental health outcomes by validating patients’ experiences and fostering therapeutic relationships that cater to individual psychological needs (52, 53). Fundamentally, patient-centered communication is driven by the expectation or need to achieve psychological health goals. From the perspective of goal-setting theory, goals are often used as self-management tools. By setting specific goals, individuals become more aware of their health needs, and these clear objectives can influence their behavioral choices, effectively promoting the development of healthy behaviors (54). It has been proven that goals have a significant promoting effect on changes in health behaviors, such as weight management, physical exercise, and healthy eating (55, 56). People achieve health goals through conscious self-management, unconscious habits, and the transition from conscious to unconscious behaviors.

At the same time, previous research has concluded that higher self-efficacy is consistently associated with improved dietary habits, increased physical activity, better stress management, and adherence to preventive health practices (57–59). Such behaviors are believed to exert their beneficial effects through multiple mechanisms, including the regulation of stress response systems, reduction of inflammatory markers, and enhancement of neurobiological processes related to mood regulation (60–62). These mechanisms collectively contribute to mitigating clinical correlates of psychological distress, such as anxiety and depression. This was congruent with previous findings.

Existing studies tend to explore the direct impact of health-related self-efficacy or healthy lifestyle behaviors on psychological distress. In this paper, by constructing a theoretical model of chain mediation, the relationship between e-health service use and psychological distress is explored. This enriches the preliminary study about health-related self-efficacy and healthy lifestyle behaviors.

Moreover, this study advances health communication theory by revealing that social media acts as a contextual moderator in the relationship between patient-centered communication and health-related self-efficacy. While prior research emphasizes the direct positive impact of patient-centered communication on health-related self-efficacy, this finding suggests that social media use can strengthen this effect, depending on the nature of online engagement. The results align with the Social Cognitive Theory, highlighting how observational learning and social reinforcement on social media may amplify the benefits of patient-centered communication—or, conversely, how exposure to misinformation or negative health narratives may diminish them. This implies that future theoretical models should integrate social media environments as dynamic factors shaping patient empowerment, moving beyond traditional clinic-based communication frameworks.

### Practical implication

This study explores the association between patient-centered communication and psychological health and contributes important value to the prevention of psychological problems. During the patient-centered dialogue process, it is necessary to enhance the health-related self-efficacy of the patients, set improvement goals for their psychological health, guide them to adopt healthier lifestyle behaviors, and thereby alleviate their psychological distress. When patients are actively involved in constructing their healthcare goals, they develop a sense of agency that mitigates feelings of helplessness and distress. Therefore, the role of goal-setting in health management cannot be overlooked. When engaging in healthy lifestyle behaviors, efforts should be made to encourage individuals to set long-term goals for improving psychological distress. Once individuals consciously set goals for healthy lifestyle behaviors, they tend to demonstrate more sustained commitment. This approach may strengthen their health-related self-efficacy and their sense of identification with a healthy lifestyle, thereby improving adherence to a healthy lifestyle, and regulating emotions.

This paper provides an important reference for the relationship between patient-centered communication and psychological distress. It has important implications for doctor-patient communication and the prevention of psychological distress. First, enhancing health-related self-efficacy is the foundation for improving an individual’s lifestyle behaviors, requiring achievable goal-setting and social support to boost their confidence. Patient-centered communication should focus on enhancing individual’s health-related self-efficacy, transforming them from “passive recipients” to “active managers” of their health. At the same time, empathy and encouragement are essential to help patients start with small steps, gradually building a sense of control and motivating sustained behavioral change. Patient-centered communication should place emphasis on empathy. Through personalized guidance, breaking down goals into manageable steps, and establishing positive feedback mechanisms, healthcare providers can help patients gradually build confidence in managing their health behaviors. Second, when individuals perceive their ability to improve lifestyle factors (such as maintaining regular exercise and balanced nutrition), they optimize physiological indicators and also alleviate health-related distress through the sense of achievement brought by behavioral changes. This creates a self-reinforcing cycle of “communication → enhanced self-efficacy → behavior improvement → distress reduction.” These findings suggest that medical professionals should integrate psychological empowerment into routine communication by employing techniques. While guiding health behavior modifications, they should simultaneously cultivate patients’ psychological resilience, ultimately achieving improvement in psychological health.

Furthermore, the moderating role of social media use in the relationship between patient-centered communication and health-related self-efficacy calls for strategic interventions by healthcare providers, policymakers, and digital platform designers. Clinicians should proactively discuss social media use during consultations, guiding patients toward credible online resources (e.g., peer support groups, and verified health influencers) to reinforce patient-centered communication. Health institutions could develop tailored social media literacy programs to help patients critically evaluate online content, mitigating misinformation risks. For policymakers, the findings underscore the need for collaboration with tech companies to promote algorithm-driven prioritization of evidence-based health content and curb harmful misinformation.

### Limitations

Several limitations of this study should be noted. First, the cross-sectional design of this study does not allow definitive causal relationships to be drawn between patient-centered communication and psychological distress. Future longitudinal studies are needed to clarify the mechanism of the mediating effect. Secondly, the study specifically examined the mediating role of health-related self-efficacy and healthy lifestyle behaviors, potentially overlooking other influencing factors. Based on this study, future research should take into account other mediators (e.g., knowledge) or moderators (e.g., health literacy and digital literacy) that significantly influence psychological distress. Third, the research findings of the current study might be impacted by sampling bias. For example, females made up approximately 56.5 percent of the respondents (N = 3535), exceeding the male sample size by 1228. Scholars should be encouraged to conduct research that collects a more representative sample to better understand the full range of e-health service use.

## Conclusion

This study provides important empirical evidence demonstrating how patient-centered communication alleviates psychological distress through two key pathways: enhancing health-related self-efficacy and promoting healthy lifestyle behaviors. Our findings reveal that patient-centered communication serves as a crucial protective factor against psychological distress, with its effects being mediated through these psychosocial and behavioral mechanisms.

Notably, the research uncovers the moderating role of social media use in the relationship between patient-centered communication and health-related self-efficacy. This suggests that social media platforms can significantly influence how effectively patient-centered communication translates into improved self-efficacy. The interaction between PCC and social media use presents both opportunities and challenges for healthcare delivery in the digital age.

These insights provide valuable guidance for healthcare providers to optimize psychological health outcomes. Future interventions should consider harnessing the synergistic effects of patient-centered communication and social media use to create more comprehensive approaches to reducing psychological distress. By understanding these complex relationships, healthcare systems can better design interventions that enhance health-related self-efficacy, encourage healthy lifestyle behaviors, and ultimately improve individual psychological well-being.

## Data Availability

Publicly available datasets were analyzed in this study. This data can be found here: https://hints.cancer.gov/data/download-data.aspx#H5C4.
